# Design, characterization and in vivo evaluation of nanostructured lipid carriers (NLC) as a new drug delivery system for hydrochlorothiazide oral administration in pediatric therapy

**DOI:** 10.1080/10717544.2018.1529209

**Published:** 2018-11-19

**Authors:** Marzia Cirri, Lavinia Maestrini, Francesca Maestrelli, Natascia Mennini, Paola Mura, Carla Ghelardini, Lorenzo Di Cesare Mannelli

**Affiliations:** a Department of Chemistry, School of Human Health Sciences, University of Florence, Florence, Italy;; b Menarini Manufacturing Logistics and Services, Florence, Italy;; c Department of Neuroscience, Psychology, Drug Research and Child Health (NEUROFARBA), Pharmacology and Toxicology Section, University of Florence, Florence, Italy

**Keywords:** Nanostructured lipid carriers, hydrochlorothiazide, hypertension, pediatric therapy, Precirol®ATO5

## Abstract

The hydrochlorothiazide (HCT) low solubility and permeability give rise to limited and variable bioavailability; its low stability makes it difficult to develop stable aqueous liquid formulations; its low dose makes the achievement of a homogeneous drug distribution very difficult. Thus, the aim of this study was to investigate the effectiveness of a strategy based on the development of nanostructured lipid carriers (NLC) as an innovative oral pediatric formulation of HCT with improved therapeutic efficacy. The performance of various synthetic and natural liquid lipids was examined and two different preparation methods were employed, i.e. homogenization-ultrasonication (HU) and microemulsion (ME), in order to evaluate their influence on the NLC properties in terms of size, polydispersity index, ζ-potential, entrapment efficiency, gastric stability, and drug release properties. Precirol®ATO5 was used as solid lipid and Tween^®^80 and Pluronic^®^F68 as surfactants, formerly selected in a previous study focused on the development of HCT-solid lipid nanoparticles (SLNs). The presence of Pluronic^®^F68 did not allow ME formation. On the contrary, using Tween^®^80, the ME method enabled a higher entrapment efficiency than the HU. Regardless of the preparation method, NLCs exhibited great entrapment efficiency values clearly higher than previous SLNs. Moreover, NLC-ME formulations provided a prolonged release, which lasted for 6 h. In particular, NLC-ME containing Tween^®^20 as Co-Surfactant showed the best performances, giving rise to a complete drug release, never achieved with previous SLN formulations, despite their successful results. In vivo studies on rats confirmed these results, displaying their best diuretic profile. Moreover, all HCT-loaded NLC formulations showed higher stability than the corresponding SLNs.

## Introduction

Children are a special category of patients. The lack of suitable specific pediatric formulations often leads to the use of extemporaneous preparations obtained starting from the dosage form for adults, by dilution of liquid formulations, opening or capsules or finely grinding of tablets, administered as suspensions. However, such manipulations give rise to a series of disadvantages, including in particular minor dosing accuracy, unknown and variable bioavailability, poor compliance for both children and caregivers, and lack of data about the preparation stability (Pandolfini & Bonati, 2005; Standing & Tuleu, 2005).

Cardiovascular drugs are widely utilized for galenic pediatric preparations in the hospital pharmacies, owing to their use for a series of clinical conditions, such as heart failure, hypertension, hypovolemic shock or edema (Associazione Ospedali Pediatrici Italiani, 2005). Among these, diuretic drugs such as hydrochlorothiazide (HCT), are often used in the management of hypertension. Owing to its low solubility and permeability (Basant Kumar Reddy & Karunakar, 2011), HCT is ranked as a BCS class IV drug (Amidon et al., 1995), and presents problems of poor and variable bioavailability (Patel et al., 1984). Moreover, stability problems of HCT in aqueous solution have been reported (Mollica et al., 1971)

At present, no liquid formulations of HCT are available on the market. Extemporaneous liquid pediatric preparations are generally obtained by suspending the drug powder in a suitable syrup (Atienza et al., 2011) or by grinding a fraction of the commercial tablets and suspending it in a liquid vehicle (Allen & Erickson, 2011). Attempts of developing stable low-dosage HCT pediatric suspensions failed, due to problems of variable drug recovery and limited stability (Santoveña et al., 2012).

Lipid-based colloidal carriers proved to be an interesting and successful strategy to enhance the stability and oral bioavailability of poorly soluble drugs. In particular, in recent years, increasing attention has been paid towards the development of the so-called solid lipid nanoparticles (SLNs), which offer an effective alternative to the classic liposomes or polymeric nanoparticles, overcoming some of their major drawbacks, such as the low stability and poor loading capacity of the first ones and the potential biotoxicity of the second ones (Müller et al., 2000; Mehnert & Mader, 2001; Mukherjee et al., 2009)

SLNs are lipid matrix composed of biocompatible and biodegradable excipients able to protect the entrapped drug, provide a controlled release and improve its bioavailability (Silva et al., 2012; Gonçalves et al., 2016). We recently developed a low-dosage liquid HCT-loaded SLN formulation for pediatric use (Cirri et al., 2017). *In vivo* studies on rats demonstrated the enhanced therapeutic efficacy of such formulation with respect to the simple drug aqueous suspension, in terms of increased diuretic effect and prolonged release.

Nanostructured lipid carriers (NLCs) represent an evolution of the original SLNs, containing a mixture of both solid and liquid lipids. The use of spatially different lipids creates imperfections in the crystal structure of the solid lipids, leading to various advantages compared to SLNs such as higher entrapment efficiency, increased physical stability, less drug expulsion during storage (Radtke & Müller, 2001; Müller et al., 2002; Radtke et al., 2005; Muchow et al., 2008; Severino et al., 2012; Beloqui, 2016).

Therefore, we considered it worthy of interest to investigate the usefulness of employing NLC as a new carrier for the oral administration of HCT in pediatric therapy, in order to assess their actual advantages compared to SLNs, in terms of drug entrapment efficiency, gastric and storage stabilities and drug release properties. With this aim, the effect of different synthetic and natural liquid lipids and of two different preparation methods, i.e. the homogenization-ultrasonication (HU) and the microemulsion technique (ME) was evaluated and compared in terms of particle size and homogeneity, ζ-potential, entrapment efficiency, stability, and drug release properties. Finally, the therapeutic efficacy of the best formulations was evaluated in vivo on rat.

## Materials and methods

### 2.1. Materials

Hydrochlorothiazide (HCT) was a generous gift of Menarini (Italy). Precirol^®^ATO5 (Glyceryl distearate/palmitostearate), Transcutol^®^HP (highly purified diethylene glycol monoethyl ether), Labrafac™ PG (propylene glycol dicaprylocaprate), Capryol™ 90 (propylene glycol monocaprylate, type II), Labrafil^TM^ Lipophile WL1349 (medium-chain triglycerides) and Labrasol^®^ (PEG-8 caprylic/capric glycerides) were kindly provided by Gattefossé (Saint-Priest, France). Oleic acid, 1-butanol, Tween^®^20 (polyoxyethylene sorbitan monolaurate) and Tween^®^80 (polyoxyethylene sorbitan monoleate) were from Merck (Darmstadt, Germany), castor and peanut oils from Galeno (Prato, Italy), sesame oil from Farvima (Firenze, Italy), sodium taurocholate from Prodotti Chimici e Alimentari S.p.A. (Basaluzzo (AL), Italy), lecithin from Carlo Erba Reagents (Milan, Italy), glycofurol (tetrahydrofurfurylether polyetylenglycol) and phosphatidylcholine from Sigma-Aldrich (St. Louis, USA). Solutol^®^HS15 (2-hydroxyethyl 12-hydroxyoctadecanoate) and Pluronic^®^F68 (Poloxamer 188) were from BASF (Ludwigshafen, Germany). Purified water was obtained by reverse osmosis (Elix^®^ Millipore, MD, USA). All other chemicals were of analytical grade.

### 2.2. Vehicles screening

#### Selection of liquid lipids

Precirol^®^ATO5 was selected as solid lipid, based on previous studies (Cirri et al., 2017). In order to select the most suitable liquid lipid for drug-loaded NLC formulation, the solubilizing power toward HCT of a series of synthetic (Transcutol^®^HP, Capryol^TM^ 90, Labrafac^TM^ PG, and Labrafil^TM^ Lipophile WL1349) and natural (oleic acid; castor, sesame, and peanut oils) liquid lipids was tested. The HCT maximum amount which can be dissolved in 5 mL of each liquid lipid was determined by adding stepwise, under stirring, increasing HCT amounts (1–200 mg) in the lipid thermostated at 65 °C, to mimic the experimental conditions needed for NLC production, and allowed to equilibrate for 24 h. HCT solubility in each system was evaluated by visual observation of disappearance of drug crystals and formation of a transparent homogeneous system (Nnamani et al., 2014).

#### Miscibility of solid and liquid lipid systems

The solid lipid Precirol^®^ATO5 was mixed with each liquid lipid in the 90:10 w/w ratio, to evaluate the miscibility of the two lipids. The mixtures were heated for 30 min at 65 °C and then allowed to cool at room temperature. Any possible sign of turbidity or phase separation process was assessed by visual inspection. Miscibility and solid state properties of cooled samples were investigated also by Differential Scanning Calorimetry (DSC). Analyses were performed with a Mettler TA4000 Star^e^ Software apparatus (Mettler Toledo, Switzerland) equipped with a DSC 25 cell, by heating samples (5–10 mg, Mettler MX5 microbalance, Mettler Toledo, Switzerland) from 25 °C to 100 °C in pierced Al pans under static air at a scan rate of 10 °C min^−1^. The instrument was calibrated for temperature and heat flow using Indium as a standard (99.98% purity; *T*_fus_ 156.61 °C; ΔH_fus_ 28.71 J g^−1^).

#### Selection of Co-Surfactants for microemulsions

Pluronic^®^F68 and Tween^®^80 were selected as surfactants, based on previous studies (Cirri et al., 2017). Selection of Co-Surfactants was performed by the titration method. Sodium taurocholate, lecithin, and phosphatidylcholine were tested as natural Co-Surfactants, and Labrasol^®^, Transcutol^®^, Glycofurol, Tween^®^20 and Solutol^®^HS15 as synthetic ones. Surfactant: Co-Surfactant mixtures (at different w/w ratios) were added to a fixed amount of a homogeneous and transparent solid: liquid lipid mixture (90:10 w/w ratio) heated at 65 °C. The resulting mixtures were progressively titrated by adding water drop to drop, under stirring, up to clouding. For each Co-Surfactant able to form microemulsions, the % range in the existence field of microemulsion was determined.

### 2.3. Preparation of NLC

NLCs were prepared according to two different methods, i.e. HU (A), and ME (B).

A) According to the HU technique (Cirri et al., 2017), the liquid lipid was added to the solid lipid Precirol^®^ATO5 melted at 65 °C. The aqueous surfactant phase containing Pluronic^®^F68 or Tween^®^80 heated at the same temperature was added to the molten lipid phase and dispersed by a T25 digital ULTRA-TURRAX homogenizer (IKA Werke, Staufen, Germany) at 10,000 rpm for 2 min. The obtained pre-emulsion was then sonicated for 3 min at 50% amplitude (Sonopuls HD 2200 sonicator, Bandelin Electronics, Berlin, Germany). Finally, the dispersion was cooled at 4 °C to solidify the lipid matrix and produce NLC. HCT-loaded NLC were prepared by incorporating the drug (200 mg) in the lipid phase to obtain a drug concentration in the final dispersion of 0.2% w/v.

B) According to the ME technique (Gasco, 1997), microemulsions were prepared by melting the solid lipid at 65 °C, together with the liquid lipid followed by the addition of the mixture Surfactant: Co-Surfactant under mechanical stirring; water was later added in a concentration range within the microemulsion existence field. The drug (200 mg) was then incorporated into the formed microemulsion. NLCs were finally obtained by adding filtered warm water to the warm microemulsion under mechanical stirring and then cooled at 4 °C to allow lipid solidification.

All samples were stored at 4 °C protected from light.

### 2.4. Characterization of NLC

#### Particle size, PDI and zeta potential

Physicochemical characterization of the lipid dispersions in terms of mean particle size, polydispersity index (PDI) and zeta potential was performed by Dynamic Light Scattering (DLS), (Zetasizer Nano-ZS90, Malvern Instruments, Malvern, UK). NLC dispersions were suitably diluted with bi-distilled water before measurement, to avoid multi-scattering phenomena. All measurements were carried out in triplicate and results expressed as average values ± S.D.

#### Drug entrapment efficiency (EE%) and loadingcapacity (LC%)

The percentage of HCT encapsulated into NLC and the loading capacity were determined indirectly by the filtration/centrifugation method (Cirri et al., 2012). Briefly, 1.5 mL of NLC dispersion were placed in the upper chamber of a membrane concentrator (Vivaspin 2, 10,000 kDa MWCO, PSE, Sartorius Stedim Biotech Ltd., Göttingen, Germany) and centrifuged for 8 min at 4000 *g* (HERMLE Labortechnik, mod. Z200A, Wehingen, Germany). The unentrapped drug collected in the filtrate in the lower chamber was assayed by UV spectroscopy (Shimadzu UV-1601) at 272.2 nm. The presence of lipids and other NLC components did not interfere with the UV assay of the drug. Entrapment efficiency (% EE) and Loading Capacity (% LC) were calculated according to Equations (1) and (2), respectively:

(1) $$EE\%=\frac{W_{initial\;drug}-\;W_{free\;drug}}{W_{initial\;drug}}\times 100$$

(2) $$LC\%=\frac{W_{initial\;drug}-\;W_{free\;drug}}{W_{lipid}}\times 100$$

where “W_initial drug_” is the initial drug amount used for NLC preparation, “W_free drug_” the amount of free drug detected in the filtrate and “W_lipid_” the total amount of lipid used.

Results are expressed as average values ± S.D. of five separate experiments.

### 2.5. Stability studies

#### Stability studies of NLC under gastric conditions

The stability under gastric conditions of NLC was tested in phosphate buffer at pH 4.5, selected as medium representing the average gastric pH value typical of infant population (Batchelor et al., 2013; Nguyen et al., 2015). Briefly, 1 mL of each NLC dispersion was put in 100 mL of pH 4.5 phosphate buffer and incubated at 37 °C up to 2 h. Samples were tested by DLS for particle size and PDI before and immediately after their introduction in the gastric buffer (*t*_0_) and at the end of the test (*t*_2h_). Determinations were performed in triplicate and results expressed as mean values ± S.D.

#### Stability studies of NLC under storage

Stability studies of NLC were performed during 3 months storage at 4 °C, by checking mean particle size, PDI and zeta potential by DLS. Visual inspection was also employed to detect any possible crystallization, precipitation, mold formation or gelling process. Determinations were performed in triplicate and results expressed as average values ± S.D.

### 2.6. *In vitro* drug release studies

*In vitro* drug release studies from NLC were performed in sink conditions according to the dialysis bag method (Cirri et al., 2012, 2017) and compared to a simple drug suspension, prepared by suspending 0.2% p/v of HCT in water under stirring. Cellulose acetate dialysis bags (Sigma-Aldrich, St. Louis, MO,USA, 12,500 cut-off) were soaked overnight in pH 4.5 gastric buffer, filled with 1 mL of NLC dispersion or suspension containing 2 mg of drug and immersed for 2 h in 100 mL of pH 4.5 phosphate buffer simulating the infant gastric pH (drug solubility in the medium about 0.7 mg/mL) and then for 4 h in 100 mL of pH 6.8 phosphate buffer (simulating the intestinal pH), thermostated at 37 °C and stirred at 50 rpm. The concentration of released drug was spectrometrically assayed at 272.2 nm at given time intervals. Each withdrawn sample from the receiver solution was replaced with an equal volume of fresh solvent. A correction for the cumulative dilution was calculated. Experiments were performed in triplicates and results expressed as mean values ± S.D.

### 2.7. Statistical analysis

The results of all the above-described studies were statistically analyzed by one-way analysis of variance (ANOVA) followed by the Student–Newman–Keuls multiple comparison post-test using the Graph Pad Prism version 6.0 software (San Diego, CA, USA). The differences were considered statistically significant when *p* < .05.

Results of in vivo studies were expressed as mean (±S.E.M.) and treated with the one-way ANOVA. A Bonferroni’s significant difference procedure was used as a *post hoc* comparison. *p*-values <.05 or <.01 were considered significant. Data were analyzed using the Origin 9 software (OriginLab, Northampton, MA, USA).

### 2.8. *In vivo* studies

For all the experiments described below, male Sprague–Dawley rats (Envigo, Varese, Italy) weighing approximately 220–250 g at the beginning of the experimental procedure, were used. Animals were housed in CeSAL (Centro Stabulazione Animali da Laboratorio, University of Florence) and used at least one week after their arrival. Four rats were housed per cage (size 26 × 41 cm); animals were fed a standard laboratory diet, tap water *ad libitum*, and kept at 23 ± 1 °C with a 12 h light/dark cycle, light at 7 a.m. All animal manipulations were carried out according to the Directive 2010/63/EU of the European Parliament and of the European Union Council (22 September 2010) on the protection of animals used for scientific purposes. The ethical policy of the University of Florence complies with the Guide for the Care and Use of Laboratory Animals of the US National Institutes of Health (NIH Publication No. 85–23, revised 1996; University of Florence assurance number: A5278-01). Formal approval to conduct the experiments described was obtained from the Animal Subjects Review Board of the University of Florence. Experiments involving animals have been reported according to ARRIVE guidelines (McGrath & Lilley, 2015). All efforts were made to minimize animal suffering and to reduce the number of animals used.

Diuretic activity was determined according to the method described by Olah et al. (2003) and Compaore et al. (2011), slightly modified. Animals were divided into 4 groups (*n* = 5) for the acute (single dose) study, with free access to food and water. Before the treatment, all animals received physiological saline (0.9% NaCl) as an oral dose of 2.5 mL/100 g body weight (Wiebelhaus et al., 1965) to impose a uniform water and salt load, and kept at room temperature. One hour later, rats were treated orally with a dose of 10 mg kg^− 1^ in the following manner: group I, serving as control, with 0.9% saline; group II with HCT suspension (0.2% p/v HCT in water); groups III, IV and V with selected NLC formulations.

A further pharmacological study was performed on two groups (*n* = 5), by treating one group with 0.9% saline (control) and the other one with empty nanoparticles, under the same experimental conditions described above.

Immediately after administration, the animals were placed in metabolic cages (one animal per cage), specially designed to separate urine and feces, and cumulative urine output at 1, 2, 4, and 6 h was recorded. The diuretic activity was expressed in mL/100 g body weight (Asif et al., 2014), while the diuretic index was calculated as the ratio between the diuresis of the animals treated with the test substance and the diuresis of the control group (Danamma et al., 2011).

## Results and discussion

### 3.1. Selection of liquid lipids

Precirol^®^ATO5 (5% w/w) was used as solid lipid for NLC preparation and Tween^®^80 and Pluronic^®^F68 (1.5% w/w) as Surfactants, since these components were formerly selected in a previous study devoted to the development of HCT-loaded SLNs (Cirri et al., 2017).

As a first step in the NLC formulation development, the solubilizing capacity of a series of synthetic (Transcutol^®^HP, Capryol™ 90, Labrafac™PG, Labrafil^TM^ Lipophile WL1349) and natural (oleic acid, castor, sesame, and peanut oils) liquid lipids toward HCT was tested, in order to select the most effective ones.

Among the examined synthetic liquid lipids, Transcutol^®^HP showed the highest solubilizing power, being the only one able to dissolve up to 200 mg of HCT in 5 mL. On the contrary, less than 80 mg HCT were dissolved in 5 mL of Capryol™ 90, Labrafac™ PG and Labrafil^TM^ Lipophile WL1349. Transcutol^®^HP was then selected as synthetic liquid lipid for NLC formulation. On the other hand, natural oils revealed a markedly lower solubilizing capacity compared to synthetic lipids. Oleic acid and castor oil were able to dissolve 10 and 5 mg of HCT in 5 mL, respectively, whereas sesame and peanuts oils solubilized only negligible amounts of drug, lower than 3 mg. Anyway, taking into account that the NLC formulation we want to develop is intended for pediatric patients, and natural oils generally being more tolerable and biocompatible than the synthetic ones, we thought it worthy of interest to consider both oleic acid and castor oil for further studies, despite their lower solubilizing power than Transcutol^®^HP.

### 3.2. Miscibility of solid-liquid lipid systems

Miscibility of solid and liquid lipid at the specific concentrations to be used in the formulation is a prerequisite for the correct development of NLC formulations (Radtke & Müller, 2001; Müller et al., 2002; Radtke et al., 2005). The miscibility between Precirol^®^ATO5 and the different liquid lipids was initially evaluated by both visual inspection upon heating and DSC analysis in different w/w ratios: 99:1, 90:10, 80:20, and 70:30, according to the ratios most commonly used in literature (Pardeike et al., 2009).

Visual inspection allowed to rule out any sign of turbidity or separation phase phenomena induced by cooling of the heated mixtures, for each liquid lipid used.

DSC studies were performed to confirm the miscibility between the selected liquid lipids and the solid lipid, based on the lowering in its melting point which would be observed following incorporation of the liquid lipid in its crystalline structure (Kasongo et al., 2011; Shah et al., 2016) as well as to gain insight about the solid state properties of Precirol^®^ATO5. The 90:10 w/w ratio was finally selected as the best compromise since it allowed to have a relatively high amount of liquid lipid, suitable to enhance the drug solubility in the lipid phase, but, in the same time, to minimize the toxicity risks.The obtained DSC curves for the binary mixture 90:10 are shown in Fig. DSC as Supplementary data).

The thermal curve of pure Precirol^®^ATO5 before the heating process exhibited the presence of a single endothermic effect with an onset and peak temperature at 50.9 °C and 58.3 °C, respectively, attributed to the melting of the stable β–polymorphic form. However, following exposure for 30 min to 65 °C and subsequent cooling at room temperature, the DSC curve of the re-solidified lipid showed the appearance of a shoulder, peaked at 48.3 °C, before the major endothermic peak at 59 °C, which may be attributed to the melting of the metastable form α-, formed during the lipid recrystallization (Saupe et al., 2005).

The decrease in onset, peak temperature and fusion enthalpy of the endothermic peak of the Precirol^®^ATO5 β–form, exhibited by each examined binary mixture, corroborated the components miscibility, at least in the considered 90:10 w/w ratio. Moreover, all solid-liquid lipid combinations showed a marked increase in the intensity of the endothermic effect at a lower temperature, observed in the DSC curve of the re-solidified lipid and related to the presence of the less crystalline α-form, and in the case of mixtures with Transcutol^®^HP this latter became even the major peak. Therefore, it appears that the incorporation of the liquid lipid into Precirol^®^ATO5 created defects within its highly ordered crystalline structure, thus favoring the transformation from the stable β- to the less ordered α-polymorphic form. (Kasongo et al., 2011). Lipid matrices with some degree of disorder are considered more suitable for formulation of nanoparticulate lipid carriers, due to their greater active ingredient payload capacity (Müller, 2002; Attama et al., 2006; Kasongo et al., 2011; Beloqui, 2016)

### 3.3. Characterization of NLC prepared by HU

The compositions of the different NLC formulations prepared by HU employing Precirol^®^ATO5 as solid lipid, Tween^®^80 or Pluronic^®^F68 as Surfactants, and three different liquid lipids are reported in Table 1. NLC formulations were characterized and compared in terms of mean particle size, PDI, zeta potential (ζ), encapsulation efficiency (EE%) and loading capacity (LC%) in order to investigate the influence of the type of surfactant and liquid lipid. The results are collected in Table 2.

**Table 1.** Composition of NLC formulations prepared with Precirol**^®^**ATO5 as solid lipid, with Tween^®^80 (NLC_1_ serial code) or Pluronic^®^F68 (NLC_2_ serial code) as Surfactant, and different liquid lipid (Transcutol (A), oleic acid (B) and castor oil (C)), both empty and loaded with hydrochlorothiazide (HCT).

Formulation	Precirol^®^ATO5(%w/w)	Surfactant type (%w/w)		Liquid lipid (%w/w)			Water (%w/w)	HCT (mg)
		Tween^®^80	Pluronic^®^F68	Transcutol	Oleic acid	Castor oil		
NLC_1A empty_	5	1.5	–	0.55	–	–	92.95	–
NLC_1A_	5	1.5	–	0.55	–	–	92.95	200
NLC_1B empty_	5	1.5	–	–	0.55	–	92.95	
NLC_1B_	5	1.5	–	–	0.55	–	92.95	200
NLC_1C empty_	5	1.5	–	–	–	0.55	92.95	
NLC_1C_	5	1.5	–	–	–	0.55	92.95	200
NLC_2A empty_	5	–	1.5	0.55	–	–	92.95	
NLC_2A_	5	–	1.5	0.55	–	–	92.95	200
NLC_2B empty_	5	–	1.5	–	0.55	–	92.95	
NLC_2B_	5	–	1.5	–	0.55	–	92.95	200
NLC_2C empty_	5	–	1.5	–	–	0.55	92.95	
NLC_2C_	5	–	1.5	–	–	0.55	92.95	200

**Table 2.** Mean particle size, polydispersity index (PDI), zeta potential (ζ), encapsulation efficiency (EE%) and loading capacity (LC%) of different NLC formulations (see Table 1 for their composition).

Formulation	size (nm)	PDI	ζ (mV)	EE%	LC%
NLC_1A empty_	571.3 ± 30.6	0.30 ± 0.02	−25.2 ± 0.4	–	–
NLC_1A_	605.6 ± 12.1	0.33 ± 0.05	−29.0 ± 0.5	73.4 ± 2.4	2.9 ± 0.3
NLC_2A empty_	367.4 ± 2.8	0.29 ± 0.02	−38.7 ± 0.9	–	–
NLC_2A_	368.4 ± 7.6	0.24 ± 0.03	−40.8 ± 0.3	53.9 ± 0.1	2.1 ± 0.1
NLC_1B empty_	483.6 ± 23.4	0.49 ± 0.03	−35.0 ± 0.6	–	–
NLC_1B_	650.6 ± 15.3	0.62 ± 0.13	−37.6 ± 0.5	79.1 ± 0.6	3.2 ± 0.3
NLC_2B empty_	–^a^	–	–	–	–
NLC_2B_	–	–	–	–	–
NLC_1C empty_	591.1 ± 22.0	0.42 ± 0.08	−35.3 ± 0.8	–	–
NLC_1C_	563.3 ± 3.7	0.36 ± 0.03	−31.6 ± 1.6	80.0 ± 0.2	3.2 ± 0.0
NLC_2C empty_	365.1 ± 1.6	0.24 ± 0.04	−42.4 ± 0.4	–	–
NLC_2C_	405.4 ± 3.8	0.22 ± 0.02	−38.8 ± 1.4	40.1 ± 3.2	1.6 ± 0.1

^a^gelification occurred.

The mean dimensions of all NLC formulations containing Pluronic^®^F68 were smaller than those of the corresponding ones with Tween^®^80, as found also by other authors (Padhye & Nagarsenker, 2013; Bhupinder & Newton, 2016), and similarly to what previously observed in the case of HCT-loaded SLNs (Cirri et al., 2017).

This finding could be probably explained with a different way of incorporation of the two surfactants in the particles outer shell (Radomska-Soukharev, 2007).

Using oleic acid as liquid lipid and Pluronic^®^F68 as Surfactant, it was not possible to obtain stable NLC dispersions, owing to gelling phenomena, which could be attributed to the building up by NLC particles of a sort of network due to lipid bridges formation among them (Freitas & Müller, 1998; Radomska-Soukharev, 2007). Such a finding may be due to the different nature of oleic acid respect to the other tested liquid lipids, being an unsaturated fatty acid. Pluronic^®^F68 itself has a natural gelling tendency, that could be accelerated by the presence of such a liquid lipid (Morishita et al., 2001).

With the same liquid lipid and Tween^®^80 as Surfactant, drug-loading led to a significantly higher PDI than the corresponding empty formulation, exceeding 0.6, suggesting the formation of a poorly uniform system, with great tendency to aggregation phenomena. Therefore, such NLC formulation was discarded.

Concerning NLC formulations containing Transcutol^®^ and castor oil as liquid lipids, regardless of the kind of surfactant used, the drug incorporation did not result in significant variations (*p* < .05) in particle dimensions, PDI and zeta potential, thus indicating in all cases the formation of homogeneous and stable nano-dispersions.

Regarding the EE%, the series of Tween^®^80-based NLC (NLC_1_) showed significantly higher values than the corresponding ones containing Pluronic^®^F68 (NLC_2_), irrespective of the type of liquid lipid used. An analogous result was observed for the loading capacity. In particular, the Tween^®^80-based NLC containing castor oil as liquid lipid showed the highest value of EE%, achieving 80%. Such a value resulted clearly higher than that obtained in our previous study for the corresponding SLN formulations (Cirri et al., 2017), and it can be reasonably attributed to the more disordered solid lipid matrix of NLC than SLNs, making them more available to incorporate more drug amounts.

### 3.4. Gastric stability studies

The gastric environment can negatively affect the stability of NLC after their oral administration. In fact, the acid pH may destabilize lipid carriers, leading to aggregation phenomena, as a consequence of attractive forces, such as Van der Waals and hydrophobic interactions (Paliwal et al., 2009; Das & Chaudhury, 2011; Luo et al., 2015) and formation of microparticles, thus negatively influencing the drug release and absorption properties of NLC (Zimmermann & Müller, 2001; Roger et al., 2009).

The gastric stability of NLC dispersions was studied in pH 4.5 buffer solution, selected as an average value for infant population, their gastric pH being less acid than that of adults, remaining in the 4–5 range after 2 h of feeding (Batchelor et al., 2013; Nguyen et al., 2015).

The difficulty of reproducing the conditions of pediatric patients *in vitro* makes the development of specific tests, able to provide appropriate biopharmaceutics tools to predict the *in vivo* performance of formulations aimed for pediatric use, a very challenging task (Batchelor et al., 2014). In this work, a lipolysis model was not used, due to the lack of specific pediatric models. In fact, the various in vitro models designed for simulating the lipolysis processes in adults are not suitable to reflect those occurring in infants, due to the differences between adults and pediatric population. The activity of digestive enzymes in the gastric and intestinal fluids of the pediatric population has been widely investigated (Zoppi et al., 1972; Hamosh et al., 1981; Hamosh et al., 1989; Boehm, 1991; Di Palma et al., 1991; Armand et al., 1996; Roman et al., 2007): in particular, concerning lipases, the human gastric lipase (HGL) was found to play an important role for the intragastric lipolysis in neonates (Moreau et al., 1988; Bernback, 1990), as well as the carboxylester hydrolase (CEH), also known as cholesterol ester lipase (CEL) and the pancreatic lipase-related protein 2 (PLRP2) among the pancreatic lipases (Johnson et al., 2013). On the contrary, the secretion of pancreatic triglyceride lipase (PTL) is lower in neonates than in adults (Fredrikzon et al., 1988; Carriere et al., 1993).

Kamstrup et al. (2017) suggested an *in vitro* model to simulate the digestion process in the pediatric gastrointestinal tract, starting from the original model developed by Zangenberg et al (2001), providing general indications about the most important physiological factors to be considered as relevant such as media volume, pH and osmolality. However, also these authors highlighted the difficulty and the complexity of reproducing *in vitro,* the *in vivo* conditions, due to both the lack of commercial availability of HGL and PLRP2 and the difficulties of estimating the levels of digestive enzymes.

The gastric stability of the samples was investigated monitoring by DLS analysis potential changes in mean particle size and PDI (as reported in Fig. gastric_stability_NLC as Supplementary data). Pluronic^®^F68-based formulations (NLC_2_ series) showed aggregation phenomena immediately after addition to the medium, followed by a further increase in mean size during the incubation period. In particular, NLC_2A_, containing Transcutol^®^ as liquid lipid showed a particle growth up to a micrometric range, together with a drastic increase in the PDI value, so this formulation was discarded. On the contrary, in the presence of castor oil as liquid lipid (NLC_2C_ formulation), the particle size, even though increased, remained in the nanometric range, and maintained a uniform size distribution.

Tween^®^80-based formulations (NLC_1_ series), differently from those containing Pluronic^®^F68, did not show any appreciable change in the mean size, probably due to a more effective steric stabilization effect of Tween^®^80, which can be ascribed to the different adsorbing pattern on particles surface exerted by the two different Surfactants (Reich, 1997; Zimmermann & Müller, 2001; Aditya et al., 2014; Luo et al., 2015,). The higher stability of Tween^®^80-based systems compared to Pluronic^®^F68 has been previously observed (Cirri et al., 2017).

However, also for NLC_1_ series, dispersions with castor oil (NLC_1C_) were more homogeneous than those with Transcutol^®^ (NLC_1A_), whose PDI rapidly increased, exceeding values of 0.6, indicative of a not uniform nanoparticle distribution.

Based on these results, only NLC formulations containing castor oil as liquid lipid and Tween^®^80 or Pluronic^®^F68 as surfactants (NLC_1C_ and NLC_2C_), which showed satisfactory stability in gastric medium, were selected for release studies in simulated gastrointestinal fluids.

### 3.5. *In vitro* release studies

**Figure 1.**
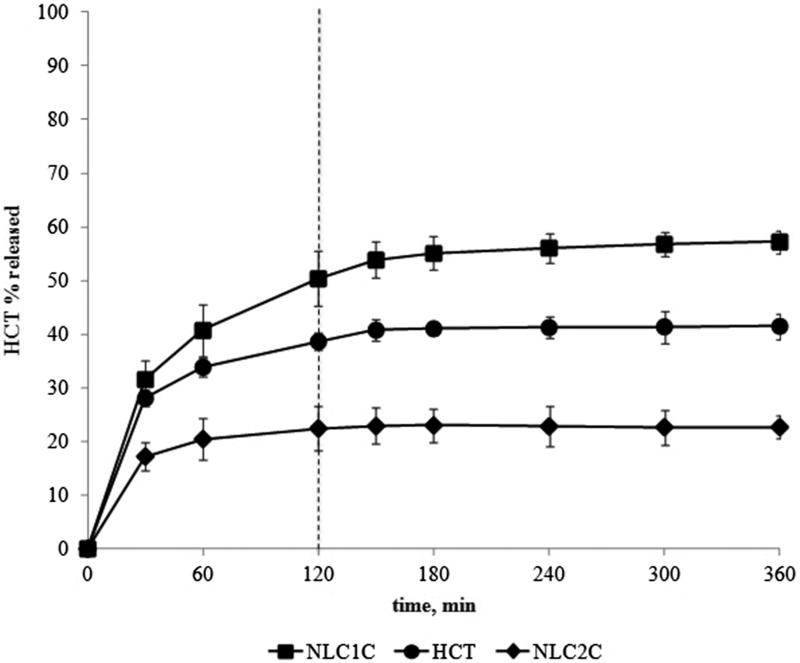
HCT *in vitro* release profiles from drug aqueous suspension or from Tween^®^80- and Pluronic^®^F68-based NLC formulations containing ricin oil as liquid lipid (NLC_1C_ and NLC_2C_) prepared by the homogenization-ultrasonication method.

The drug release curves obtained from the selected NLC formulations prepared by HU (NLC_1C_ and NLC_2C_) are shown in Figure 1 together with that of the simple drug aqueous suspension for comparison purpose. The drug suspension exhibited a quite fast release rate in gastric medium achieving nearly 40% drug released, being the fraction of drug in solution immediately ready to diffuse. Such a % value remained almost constant during the next 4 h in pH 6.8 buffer. As previously observed for HCT-based SLNs (Cirri et al., 2017), the type of surfactant clearly influenced the drug release profile. For NLC containing Pluronic^®^F68 (NLC_2C_) a slowing down in the drug release rate was observed, compared to the simple drug suspension, achieving values only slightly higher than 20% drug released at the end of the text. On the contrary, Tween^®^80-based NLC (NLC_1C_) showed a better drug release profile with respect to the simple drug suspension, allowing to achieve nearly 60% drug released. However, a complete drug release was not achieved. Therefore, a new series of NLC formulations were prepared, by using the ME method.

### 3.6. Preparation and characterization of microemulsion-based NLC

A first screening aimed to select the Co-Surfactants able to form microemulsions in the presence of a fixed amount of Precirol^®^ATO5 and castor oil (90:10 w/w ratio) with each Surfactant (Pluronic^®^F68 and Tween^®^80), was performed by the titration method with water.

The amounts of Surfactant, solid and liquid lipid were fixed at the same concentration as in the final NLC composition prepared by HU (NLC_1C_ and NLC_2C_). A 1:1 w/w Surfactant: Co-Surfactant (S:Co-S) ratio was initially used.

In the presence of Tween^®^80 as Surfactant, neither sodium taurocholate nor lecithin, phosphatidylcholine or Labrasol^®^ allowed microemulsions formation, whereas Transcutol^®^, Glycofurol, Tween^®^20 and Solutol^®^ HS gave rise to clear empty microemulsions. However, the loading of the drug was not possible with these Co-Surfactants, except for Solutol^®^ HS which enabled obtainment of transparent drug-loaded microemulsions when used in a % ranged between 11–17% w/w of the total microemulsion composition.

On the other hand, in the presence of Pluronic^®^F68 as Surfactant, only Tween^®^20 and Solutol^®^ HS permitted the formation of empty microemulsions, but none of them allowed to obtain drug-loaded microemulsions.

Only a few of the tested S: Co-S combinations were successful, since diluting with water and cooling lead to microemulsion breakage, converting it into nanoemulsion, where the formation of lipid particles occurred upon recrystallization of the lipid phase. The nanoemulsion formation and the lipid particle size are affected by several variables, including the microemulsion composition (Chaturvedi & Kumar, 2012).

Therefore, we evaluated the possibility of extending the number of Co-Surfactants able to form stable drug-loaded microemulsions by increasing their content in the S:Co-S systems based on literature data about their safety and toxicity (Food Safety Commission, 2007; Atthaporn et al., 2011; Gopinathan et al., 2013; Sullivan et al., 2014). However, among the Co-Surfactants resulted able to form empty microemulsions, only Glycofurol and Tween^®^20 allowed their use at 1:2 and 1:4 w/w S: Co-S ratios.

Nevertheless, Glycofurol was not able to form drug-loaded microemulsions neither in 1:2 nor in 1:4 w/w S: Co-S ratios, regardless of the type of Surfactant used.

On the contrary, Tween^®^20 permitted the obtainment of drug-loaded microemulsions both in the 1:2 and 1:4 w/w ratios if used, respectively, in the 22–28 and 33–44% range of the total microemulsion composition, with Pluronic^®^F68 as Surfactant, whereas only in the 1:4 w/w ratio with Tween^®^80, if used in the 29–44% range.

Based on these results, a new series of NLC was prepared starting by microemulsions containing S:Co-S mixtures of Tween^®^80:Tween^®^20 at 1:4 w/w ratio (NLC_I_), Tween^®^80:Solutol^®^HS at 1:1 w/w ratio (NLC_II_), Pluronic^®^F68:Tween^®^20 at 1:2 and 1:4 w/w ratios (NLC_III_ and NLC_IV_).

The microemulsion: water v/v ratio was varied, depending on the % of Co-Surfactant used, in order to keep the final concentration of solid and liquid lipid and Surfactant constant (5%, 0.55%, and 1.5%, respectively).

The obtained systems were characterized in terms of particle size, PDI, zeta potential, encapsulation efficiency and loading capacity (Table 3).

**Table 3.** Mean particle size, polydispersity index (PDI), zeta potential (ζ), Encapsulation Efficiency (EE%) and Loading Capacity (LC%) of different ME-based NLC formulations.

Code	S:Co-S (w/w ratio)	size (nm)	PDI	ζ (mV)	EE%	LC%
NLC_I_	Tween^®^80:Tween^®^20	327.6 ± 3.7	0.38 ± 0.03	−25.1 ± 0.3	93.2 ± 0.5	3.7 ± 0.2
	1:4					
NLC_II_	Tween^®^80:Solutol^®^HS	429.3 ± 11.2	0.39 ± 0.02	−25.0 ± 0.9	86.0 ± 0.3	3.4 ± 0.2
	1:1					
NLC_III_	Pluronic^®^F68:Tween^®^20	>1μm	1	–	–	–
	1:2					
NLC_IV_	Pluronic^®^F68:Tween^®^20	>1μm	1	–	–	–
	1:4					

The Pluronic^®^F68-based microemulsions did not give rise to stable NLC formation; in fact, both kinds of dispersed systems (NLC_III_ and NLC_IV_) showed particle size values in the micrometric range, with very high PDI values. On the contrary, in the presence of Tween^®^80, both with Tween^®^20 (NLC_I_) and Solutol^®^HS (NLC_II_) as Co-Surfactants, stable NLC were obtained with particle size values around 330 and 430 nm, respectively, which resulted smaller than those of NLC obtained with the HU method with the same Surfactant.

Furthermore, NLC prepared by the ME technique exhibited higher EE% values than the corresponding Tween^®^80-based NLC produced by the HU method, allowing to achieve values ranging from 86% with Solutol^®^ HS up to 93% with Tween^®^20 (vs 80% obtained with the HU method). These EE% values were also clearly higher than those obtained with the best SLN formulation (57.5%) (Cirri et al., 2017).

Both NLC_I_ and NLC_II_ formulations were stable in pH 4.5 gastric buffer, without showing any significant change in mean size and PDI both after addition to the medium and during the incubation period (see Fig. gastric_stability_NLC-ME reported as Supplementary data).

**Figure 2.**
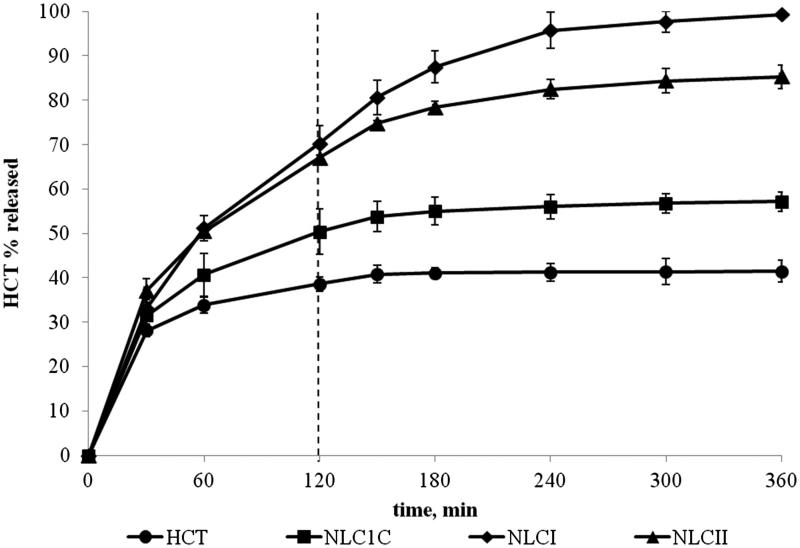
HCT *in vitro* release profiles from NLC formulations produced by the microemulsion method containing S:Co-S mixtures of Tween®80:Tween®20 at 1:4 w/w ratio (NLC_I_) and Tween®80:Solutol®HS at 1:1 w/w ratio (NLC_II_) in comparison with the corresponding Tween^®^80-based NLC formulations prepared by the homogenization-ultrasonication method (NLC_1C_) and simple aqueous suspension.

In vitro release studies (Figure. 2) revealed a prolonged release, which lasted for 6 h from both the formulations, higher not only than the simple HCT aqueous suspension but also than the corresponding NLC prepared with the HU method (NLC_1C_). NLC_II_ achieved 80% drug released at the end of the test.

Moreover, NLC_I_ showed in absolute the best performance, giving rise to a complete drug release within 6 h, never achieved with the previous SLN formulations (Cirri et al., 2017). Such a finding confirmed the microemulsion technique as the most effective method to produce HCT-loaded NLC formulations.

### 3.7. *In vivo* studies

**Figure 3.**
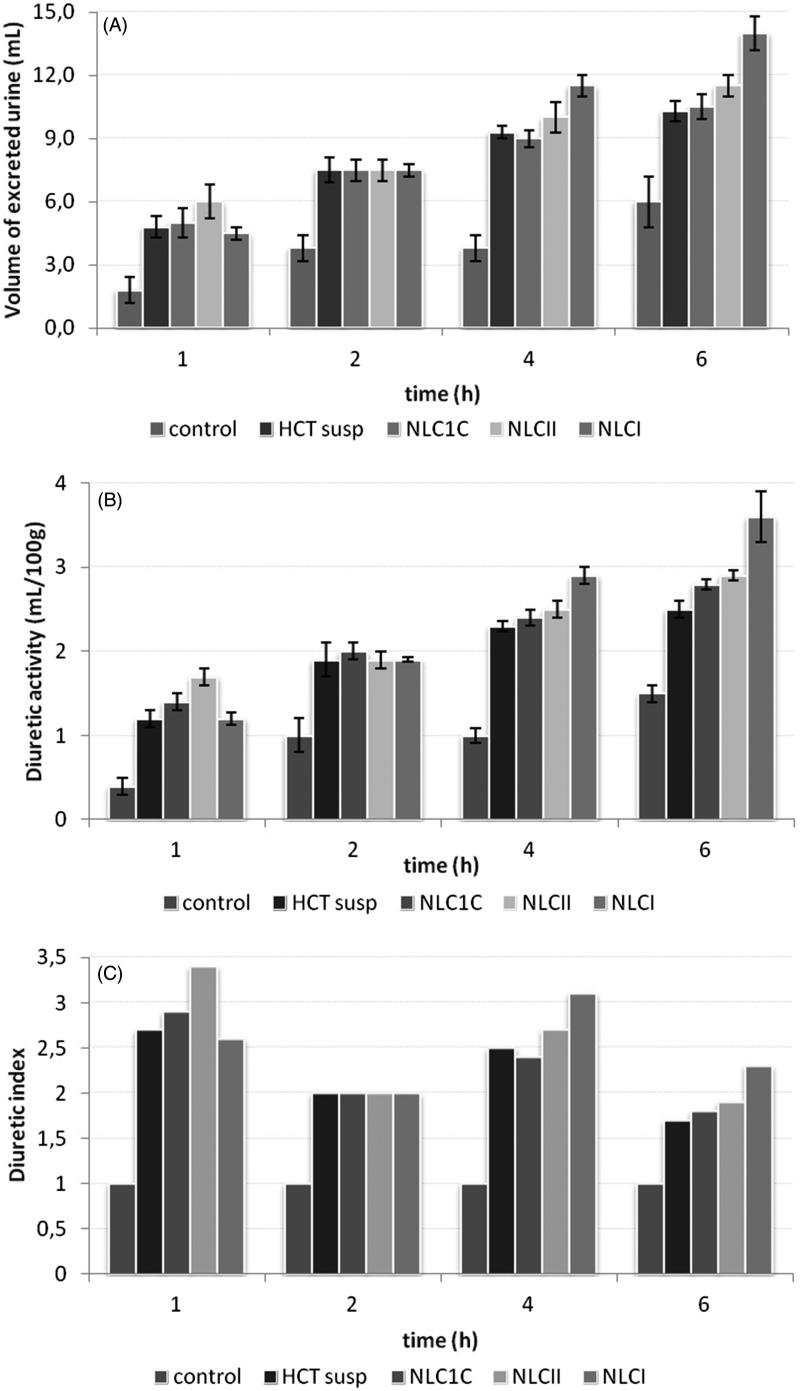
Diuretic effect expressed in terms of: volume of excreted urine (mL) (A); diuretic activity (ml/100g body weight) (B) and diuretic index (C) after oral administration (drug dose of 10 mg kg^−1^) of the selected HCT-loaded NLC_I_, NLC_II_ and NLC_1C_ formulations.

The diuretic effect in terms of volume of excreted urine, diuretic activity, and diuretic index after oral administration of the selected HCT-loaded NLC_I_, NLC_II,_ and NLC_1C_ formulations (all at the drug dose of 10 mg kg^−1^) was evaluated in normal adult male rats and compared with the effect induced by oral administration of the same dose of HCT as aqueous suspension (Figure 3 (A–C)). As can be observed in Figure 3(A), HCT suspension, acutely administered, significantly increased the diuresis from 1 h up to 6 h after treatment in comparison to the control group treated with saline (4.8 ± 0.5 ml, 10.3 ± 0.5 ml *vs* 1.8 ± 0.6 ml, 6.0 ± 1.2 ml). All the tested NLC formulations were able to significantly increase the urinary outflow up to 6 h after administration in comparison to the control group. However, treatments with NLC_1C_ showed a urinary output comparable to that evoked by HCT suspension. An appreciable improvement in the excreted urine volume after 4 and 6 h was instead for observed in the case of NLC_II_. A further increment of the urine volume was obtained with NLC_I_ formulation, which displayed the better diuretic profile, significantly (*p* < .05) superior than that of HCT suspension at 4 h–6 h after treatment.

The diuretic activity, expressed as ml/100g body weight, highlighted similar results to those above described (Figure 3 (B)). All compounds were able to increase the diuretic activity up to 6 h after treatment in comparison to the control group, showing an analogous trend of efficacy: NLC_I_ > NLC_II_ > NLC_1C_ ≈ HCT, further confirming the better efficacy of NLC_I_ treatment.

At last, the diuretic index was calculated as the ratio between the diuresis of the animals treated with the test compound compared to the diuresis of the control group (Figure 3C). Also, in this case, NLC_I_ was the most effective formulation, exhibiting the highest diuretic index at 4 h and 6 h than all the others treated group.

The further pharmacological study performed on rats treated with empty NLC demonstrated that the components of the nanoparticles have no effect on diuresis, compared to the control, showing similar values (*p* ≫ .05) of both volumes of excreted urine and diuretic activity, leading to a diuretic index equal to 1.

Therefore, we can conclude that *in vivo* studies demonstrated that the HCT formulation as NLC_I_ led to a superior therapeutic efficacy in terms of increased intensity of the diuretic effect with respect to the simple aqueous drug suspension attributable to an enhancement of drug bioavailability. Moreover, an extension of the duration of action can be reasonably assumed, based on the sustained release properties exhibited by the developed NLC_I_ formulation.

In order to better highlight the overall effect of each treatment, the AUC values (the area under the urine volume curve versus time) obtained by each drug treatment were calculated. Their comparison confirmed the increased bioavailability of HCT when administered as NLC prepared with the microemulsion method: in particular, the most important enhancement of the oral bioavailability was observed for NLC_I_ formulation, whose AUC values resulted almost 20% higher than that of the corresponding drug suspension.

### 3.8. Stability studies of stored NLC

Stability studies of the selected NLC formulations, stored at 4 °C, were carried out over 3 months and the results expressed in terms of mean particle size, PDI and zeta potential.

A moderate reduction of the particle size was registered for all formulations during the storage period. Such a trend had been already observed in a previous work (Cirri et al., 2017) and attributed to some loss of lipid (Radomska-Soukharev, 2007), depending on the type of Surfactant, that can lead to a different incorporation in the nanoparticles surface, or to a different solubilization capacity for water in the lipid phase, thus differently affecting the lipid matrix chemical stability (Radomska-Soukharev & Müller, 2006).

NLC_1C_ formulation was found to be stable up to 2 months, since the marked increase in the PDI observed after this time (over 0.6) could indicate a polydisperse system with a tendency to aggregation. On the contrary, in the case of ME-based NLC, no important variations of PDI values (estimated about 0.4 for freshly prepared NLC) were observed for all NLC series during the considered storage period of three months_._ Both NLC_I_ and NLC_II_ showed zeta potential values lower than 30 mV but sufficient to obtain stable dispersions due to the presence of a sterically stabilizing surfactant (Radomska-Soukharev, 2007).

HCT-loaded NLC prepared by the ME method resulted stable for a longer time than the corresponding previous SLNs (Cirri et al., 2017), thus evidencing the advantage of NLC compared to SLNs also in terms of improved storage stability.

## Conclusions

In this study, new low-dosage liquid oral formulations of HCT were developed, by using NLC as carrier delivery system.

The study highlighted the importance of the proper choice of the NLC components and of the preparation method on their performance. A different influence of the preparation method on both the technological and release properties of NLC as well as on their stability was also pointed out. The ME technique proved to be the most effective method to produce HCT-loaded lipid formulations, giving rise to advantages in terms of smaller particle size (ranging from about 300 and 400 nm for NLC_I_ and NLC_II_ obtained by microemulsion, vs more than 500 nm for NLC_1C_) and increased entrapment efficiency, allowing to achieve value of 93% and 86% EE, respectively. Such values resulted higher not only compared to NLC obtained by HU method but also to the previously prepared SLNs (57.5%). (Cirri et al., 2017).

In particular, NLC_I_ was the best formulation_,_ assuring a complete and sustained drug release up to 6h (never achieved with the previous SLN formulations (Cirri et al., 2017), that resulted in an improved therapeutic efficacy of this formulation in terms of increased diuretic effect, when orally administered in rats, compared to the simple aqueous drug suspension.

Finally, all the HCT-loaded NLC formulations prepared by the ME method showed stability over three months, higher than the previous corresponding SLN formulations (Cirri et al., 2017).
